# Multiscale Regulation of the Intervertebral Disc: Achievements in Experimental, In Silico, and Regenerative Research

**DOI:** 10.3390/ijms22020703

**Published:** 2021-01-12

**Authors:** Laura Baumgartner, Karin Wuertz-Kozak, Christine L. Le Maitre, Francis Wignall, Stephen M. Richardson, Judith Hoyland, Carlos Ruiz Wills, Miguel A. González Ballester, Michael Neidlin, Leonidas G. Alexopoulos, Jérôme Noailly

**Affiliations:** 1 BCN MedTech, Department of Information and Communication Technologies, Universitat Pompeu Fabra, 08018 Barcelona, Spain; laura.baumgartner@upf.edu (L.B.); carlos.ruiz@upf.edu (C.R.W.); ma.gonzalez@upf.edu (M.A.G.B.); 2 Department of Biomedical Engineering, Rochester Institute of Technology (RIT), Rochester, NY 14623, USA; kwbme@rit.edu; 3 Schön Clinic Munich Harlaching, Spine Center, Academic Teaching Hospital and Spine Research Institute of the Paracelsus Medical University Salzburg (Austria), 81547 Munich, Germany; 4 Biomolecular Sciences Research Centre, Sheffield Hallam University, Sheffield S1 1WB, UK; c.lemaitre@shu.ac.uk; 5 Division of Cell Matrix Biology and Regenerative Medicine, School of Biological Sciences, Faculty of Biology, Medicine and Health, University of Manchester, Manchester Academic Health Sciences Centre, Oxford Road, Manchester M13 9PT, UK; Frankie_wignall90@hotmail.co.uk (F.W.); S.Richardson@manchester.ac.uk (S.M.R.); judith.a.hoyland@manchester.ac.uk (J.H.); 6 Catalan Institution for Research and Advanced Studies (ICREA), Pg. Lluis Companys 23, 08010 Barcelona, Spain; 7 Department of Mechanical Engineering, National Technical University of Athens, 15780 Athens, Greece; michael.neidlin@gmail.com (M.N.); leo@mail.ntua.gr (L.G.A.)

**Keywords:** intervertebral disc, extracellular matrix, disc cell molecular biology, multifactorial cell stimulation, intervertebral disc degeneration, regenerative medicine, multiscale modeling, computational multiphysics, computational systems biology

## Abstract

Intervertebral disc (IVD) degeneration is a major risk factor of low back pain. It is defined by a progressive loss of the IVD structure and functionality, leading to severe impairments with restricted treatment options due to the highly demanding mechanical exposure of the IVD. Degenerative changes in the IVD usually increase with age but at an accelerated rate in some individuals. To understand the initiation and progression of this disease, it is crucial to identify key top-down and bottom-up regulations’ processes, across the cell, tissue, and organ levels, in health and disease. Owing to unremitting investigation of experimental research, the comprehension of detailed cell signaling pathways and their effect on matrix turnover significantly rose. Likewise, in silico research substantially contributed to a holistic understanding of spatiotemporal effects and complex, multifactorial interactions within the IVD. Together with important achievements in the research of biomaterials, manifold promising approaches for regenerative treatment options were presented over the last years. This review provides an integrative analysis of the current knowledge about (1) the multiscale function and regulation of the IVD in health and disease, (2) the possible regenerative strategies, and (3) the in silico models that shall eventually support the development of advanced therapies.

## 1. Introduction

The intervertebral disc (IVD) is a major mechanical load-bearing organ and is responsible for the functional articulation of the spine. It is composed of three tissues: the nucleus pulposus (NP), the annulus fibrosus (AF), and the cartilage endplate (CEP) that strongly interact among each other. These interactions depend on the composition and ultrastructure of each tissue that are largely regulated by the response of the disc cells to microenvironmental biological, chemical, and physical cues, transmitted to the cells through the extracellular matrix (ECM). A balance between anabolic and catabolic processes on a cellular level, i.e., tissue homeostasis, is essential for a healthy turnover of the ECM components and optimal aging [1,2,3,4]. In contrast, the perturbation of this equilibrium might cause IVD degeneration (DD), with elevated catabolic activity leading to disease progression [1,5,6,7].

The first morphological signs of DD might appear already during adolescence and largely progress toward moderate to advanced degeneration within the three next decades of life [8]. Such progression is manifest in MRI images [9], visible though a general reduction of the disc height; a shift of image signal in the NP toward inhomogeneous, gray or black shaded nuances that reflect dehydration and cracks; a loss of distinction between the NP and AF regions; and possible endplate defects and disc bulging or herniation [9,10,11]. It is commonly believed that these changes start with a drop of pressure within the NP because of dehydration.

On the one hand, the drop of intradiscal pressure makes the axial deformation of the disc increase under the action of external mechanical loads, which eventually favors the collapse of the AF structure. The AF lamellae become unorganized, fissured, and used to bulge within the NP and/or outward [12]. On the other hand, water loss is commonly interpreted as a consequence of proteoglycan (PG) depletion [2]. Interestingly, theoretical simulations associated the loss of PG and tissue swelling with the propagation of radial crack formation [13], leading to radiating annular tears [14], which might end up in IVD herniation.

Though appealing, such a systematic explanation of DD progression explains only a subset of herniated IVD phenotypes [15], i.e., the AF-driven phenotype of DD. Even though, the spatiotemporal emergence of several subsets of phenotypes related to different types of AF tears [14,16] remains difficult to explain. Furthermore, endplate-driven DD is also recognized as a source of important disc disease phenotypes [17]. Signs of inflammation around the endplate, called Modic changes, are often visible [18] and have been associated with DD [19], with endplate defects and with severe low back pain (LBP) [20,21]. Yet, the associated pathophysiology remains largely unexplained. Interestingly, the understanding of IVD-related diseases differs between authors, which led to an attempt to standardize the nomenclature about normal and pathological lumbar disc by the “Combined Task Forces of the North American Spine Society” [22]. In any case, degenerative disc changes at the tissue level alter the mechanics of the entire IVD [12] and account for at least 40% of all LBP cases [23,24].

The IVD architecture provides the non-degenerated IVD with adequate resistance to traumatic loading, as seen in sport or traffic accidents, such that isolated, traumatic IVD ruptures are hardly seen [25]. Disc rupture is, therefore, widely accepted to be a slow process, consisting of an accumulation of micro injuries under rather physiological loads, promoted by intricate biochemical and mechanobiological processes that end up in debilitated tissues. Considerable progress has been made over the past 20 years identifying risk factors for DD, and the condition, long thought to be secondary to occupational loading, has also been shown to be highly heritable [26,27]. Yet, heredity was confirmed to be significant (55%) for a reduced number of phenotypes such as endplate defects [20] and explains less than 50% of the progression of DD in the lower lumbar spine [26] where mechanical loads are the highest. More recently, greater understanding of the interplay between genes, cellular behavior, and mechanobiology has been achieved [28], and a causal link has been proposed between IVD pathology and the expression of cytokines and of structural protein proteases from resident cells [29,30,31,32,33].

The understanding of the multiple interplays within the IVD is further challenged by the need to consider the delicate nutritional balance to ensure cell survival and activity [34] in what is the largest avascular organ of the human body. Controlling inflammation, nutrition, mechanical deformations, and the interactions thereof appears cornerstone, therefore, to understand where to act, to slow down, stop, or reverse DD through molecular or cellular therapies or biomaterial-based strategies. Current literature provides a wealth of information about the response of IVD cells to inflammatory, nutritional, and mechanical isolated stimuli. Yet, such knowledge is not sufficient to apprehend and control the complex combination of factors that effectively shape the microenvironment of disc cells in situ. The comprehensive understanding of the emergence and net effect of multiple combinations of cell stimulators is difficult to achieve through experimental and/or clinical observations. Fortunately, computational implementations of theoretical mechanical, multiphysics, and biology models are constantly growing, and simulations reveal unsuspected capacity to reasonably predict multifactorial tissue or ECM regulation at different scales [35,36] or specific degeneration paths [37].

Accordingly, this review aimed to provide an overview of the latest findings about the IVD function and regulation in health and disease at the tissue, cell, and molecular levels; about progresses in IVD regenerative medicine; and about in silico research for knowledge integration and discovery over different time and length scales.

## 2. IVD Extracellular Matrix in Health and Disease

**Figure 1:**
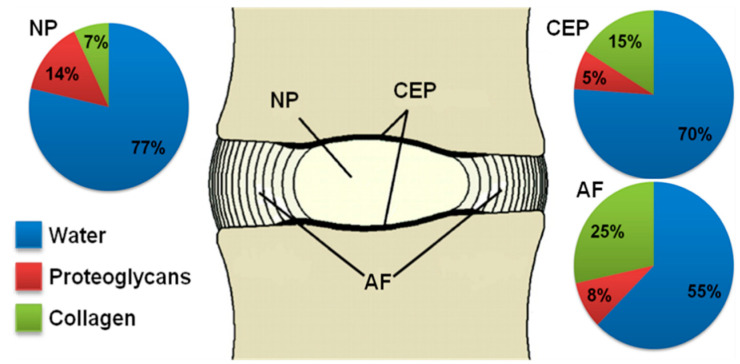
Biochemical composition of disc tissues ([46], adapted from [47]).

The biochemistry and the ultrastructure of the intervertebral disc ECM regulate the physical interactions among the disc tissues, i.e., the CEP, the AF, and the NP, and provide the IVD with unique mechanical functions [38,39]. The main ECM components of the IVD tissues are water, collagen (types I and II), and PG, and the relative contents and organization of these components are finely tuned in each disc tissue (Figure 1) [38,40].

The solid matrix of the NP mostly contains PG and non-oriented collagen type II, while the CEP contains PG and highly oriented collagen type II [41]. The AF is made of concentric bundles of collagen types I and II. While collagen type I is predominant, the relative amounts of collagen type II increase from the outer AF to the inner AF, adjacent to the NP [42]. PG is present in the interlamellar space, along with small amounts of elastic fibers and other types of fibrils [43]. It has been demonstrated that the turnover rate of collagen and aggrecan in the IVD is relatively slow due to long half-lives, i.e., around 95 and 12 years, respectively [44,45].

Interestingly, collagen turnover rate decreases with age, along with increased synthesis of types I and III collagen [3], whereas the turnover rate of aggrecan increases, the result being a gradual progression of a more fibrotic and less hydrated tissue with increasing age. In the following subsections, the current knowledge about the main ECM components’ PG, collagens, and water is summarized.

### 2.1. Proteoglycans

Aggrecan is the principal PG inside the IVD. Glycosaminoglycan (GAG) chains are attached to the main core protein of the PG, and they contain negatively charged sulphated groups [48]. The size and aggregation of PG molecules impedes these negative charges to move spatially and, because tissue electro-neutrality needs to be respected, small counter-ions, e.g., potassium and sodium, are attracted from the interstitial fluid, generating a gradient of chemical potentials between the regions respectively internal and external to the disc. Such a gradient can only be reduced through the entrance of water in the IVD, known as Donnan osmosis. The tissues swell and the collagen fibers become tensed, leading to an intradiscal osmotic pressure. Osmotic pressurization and IVD hydration are crucial for the basal hydration of the NP and the functional biomechanics of the IVD.

With age or degeneration, the total content of PG decreases in all disc tissues [49,50], and increased amounts of small non-aggregating PG are found [49,51]. In the NP, the drop of PG content and non-aggregating PG negatively affect the osmotic potential and the capacity of the tissue to attract water. Whether the loss of PG affects the mechanical stability of the IVD at the macroscopic level remains unclear [52], but theoretical approaches suggest that it favors the initiation and propagation of radial cracks through the IVD [13], as observed in IVD specimens [14]. Furthermore, the accumulation of non-aggregating PG might favor the transport of small glycans out of the disc during daily load cycles [41], and IVD glycoprofiles might be a hallmark of DD [53]. High-weight hyaluronan-based molecules were pointed out as potential protectors against DD [54], whereas hyaluronic acid fragments would increase the expression of key inflammatory cytokines by IVD cells [55]. Interestingly, during normal aging the IVD shows less than 1% height loss per year, whereas degenerating discs loose approximately 3% height per year [17].

In the AF, PG is mostly present in interlamellar spaces. On the one hand, these interlamellar spaces have been identified as the preferred path for the extrusion of nuclear material [56]. On the other hand, single nucleotide polymorphism associated to the aggrecanase *ADAMTS 5* has been significantly associated with AF tears in DD [57], suggesting a relevant implication of interlamellar PG in the IVD pathophysiology. In the CEP, the control of the mobility of molecules by PG probably ensures key protection against the loss of structural proteins and water through the bony endplates [58]. While PG depletion with age or DD might explain the increased permeability of the CEP measured with aging [59], in silico models suggest that the fine-tuning of PG contents within the CEP is actually important to avoid critical chronic dehydration of the IVD under daily mechanical loads [37].

### 2.2. Collagen

Collagen types II and I are the major structural component of the IVD. Type I collagen forms highly oriented concentric lamella within the AF, which provides the AF with resistance to multiaxial loads and finely tunes the mechanical strength of the disc. Collagen type II forms a loose network, especially within the NP, and it is more extensible than collagen type I [60,61].

In the NP, the flexibility of collagen type II network allows the swelling of the PG through Donnan osmosis, whereas the elastic response of the stretching fibers generates tissue turgidity, providing the IVD with strength under high pressurization and relative flexibility otherwise. The first oriented collagen bundles appear oriented in the transition zone between the NP and the AF and the proportion of collagen type I to collagen type II increases toward the outer AF [42]. The presence of collagen type II would limit the lateral aggregation of collagen type I fibrils and leads to matrices of increased porosity [62], which fosters proper hydration of the inner IVD and the transport of molecules to disc cells. At the same time, the increasing amount of collagen type I toward the outer AF increases the effective resistance to fluid flow, favoring hydrostatic pressures and proper cell phenotypes in the inner AF [63]. The CEP matrix has predominantly collagen type II fibers. While it needs to allow the transport of important solutes between the disc and the bone marrow [64], its hydraulic permeability, of the order of 1.10^−14^ m^4^/Ns [65], is one order of magnitude lower than the radial permeability of the AF [66]. Interestingly, piezoelectric potentials associated with the disc collagen fibers have been measured and were suggested to be one of the triggers of functional cell alignments, especially in the AF [67]. In general, it is clear that the functional distribution of collagen types I and II throughout the IVD supports multiple important functions.

With degeneration or aging, the relative amount of collagen type I increases in the NP [8], which in addition to PG depletion would also explain the dehydration of the IVD, i.e., reductions of tissue porosities [68], in general. Decreased tissue porosity due to modified balance between collagen types I and II would explain why water and PG contents only moderately correlate to each other [69]. Collagen cross-links are also important; within the normal NP, high concentrations of pyridonoline cross-links are found, but with degeneration these cross links are replaced with the pentosidine cross-links [70,71], which increase the susceptibility of the tissue to tears [71,72].

Other collagens such as types III, V, VI, IX, and XI make up around 20% of the total collagen components of the disc and are thought to be involved in the organization of the collagen fibrils [73] and to play a key role in the functional mechanical behavior of the complex AF interlamellar regions [43]. Remarkably, type VI collagen is an important pericellular molecule [74] thought to be essential for the mechanosensing of IVD cells [75,76,77].

### 2.3. Water

As discussed in Section 2.1 and Section 2.2, the function of the IVD tissue matrices cannot be dissociated from the very specific interactions of the ECM components with interstitial water. Remarkably, the physics of water in the IVD and the macroscopic effect thereof depend on the balance of PG and collagen contents. While PG controls Donnan osmosis, it might further affect the shear stiffness of cartilage-like tissues [78]. Multi-physics models and experiments also suggest that the effective turgidity of disc tissues is additionally controlled by the existence of a dual porosity, generated by volumes of exclusion of PG molecules generated by the fibrillar matrix [79,80].

## 3. IVD Cell Activity and Molecular Biology in Health and Disease

The cells responsible for disc maintenance represent only 1% of the volume of the organ [81], the disc cell densities (~4 × 10^3^ cells/mm^3^ in the NP; ~9 × 10^3^ cells/mm^3^ in the AF; ~15 × 10^3^ cells/mm^3^ in the CEP) being among the lowest within the body, due to the low nutrient supply [82]. These densities decrease with aging and DD [82,83,84]. The cells of the CEP are chondrocytes [82], while those in the outer AF are similar to fibroblasts. The NP cells of a mature human disc are spherical and, while similar to chondrocytes [85], they synthesize a greater proportion of PG than chondrocytes, with a PG-to-collagens ratio of about 27:1 [86], and have a number of distinctive cell markers [87,88]. Mature NP cells are uniquely derived from notochordal cells, which, in humans, are lost during adolescence [89,90,91]. From one IVD tissue to another, cells display transitional phenotypes, illustrating the likely influence of their microenvironment on their phenotype and activity [63].

### 3.1. Multifactorial Regulation of Cell Activity in Health

The IVD has low nutritional supply with blood vessels located in the vertebral endplates and outer AF [82]. This leads to a hostile environment for cells, characterized by low oxygen tension, low glucose concentrations, high lactate levels, i.e., low pH, and high osmolality, altogether under the action of dynamic loads [92,93,94,95,96,97]. However, the cells of the IVD are remarkably adapted to such conditions [98].

In hypoxia, the increased production of lactate generated during adenosine triphosphate synthesis decreases the pH. Accordingly, IVD cells express a number of control mechanisms that maintain pH homeostasis, such as expression of plasma membrane monocarboxylate transporters [99] and bicarbonate recycling mechanisms [100]. NP cells further show robust and constitutive hypoxia inducible factor (HIF) 1 expression, and under hypoxic conditions the inducible subunit of HIF-1, HIF-1α, accumulates due to the inhibition of prolyl hydroxylase enzymes. Then, it translocates to the nucleus, where it binds to the constitutively expressed subunit HIF-1β. Subsequent binding of this dimer to hypoxia response elements on the promoter region of target genes allows the regulation of gene expression [101]. HIF-1α has been shown to contribute to the survival of NP cells in the harsh, hypoxic environment by increasing gal-3 expression, thereby inhibiting Fas receptor/Fas ligand-mediated apoptosis [102]. It may also be involved in hypoxia-driven suppression of NP cells’ autophagy via inactivation of the mTOR (mammalian target of rapamycin) signaling pathway [103]. Furthermore, HIF-1 seems to play a crucial role in supporting adequate energy metabolism (i.e., anaerobic glycolysis) in NP cells by regulating the expression of the glucose transporters GLUT-1, GLUT-3, and GLUT-9 [104]. The crucial role of HIF-1α in NP homeostasis has been underlined by knockout experiment in mice, whereby HIF-1α deficiency resulted in DD, as evidenced by reduced PG and collagen II contents [105]. Interestingly, research in other areas points toward cross talk between the nuclear factor kappa B (NF-κB) and HIF-1 signaling pathways [106], which could constitute a molecular link between hypoxia and inflammation. First data in NP cells support this notion as prolyl hydroxylase domain-containing protein 2, able to degrade HIF-1α [107], was shown to co-activate NF-κB signaling (Section 4.1) [108].

ECM osmolarity fluctuates (~430 to 496 mOsm ) with normal daily activity in the IVD [109,110] and disc cells are well adapted to respond to these fluctuations [111,112] through robust expression of osmosensitive transcription factor TonEBP (tonicity-responsive enhancer binding protein),which maintains cellular function under daily osmotic changes [113,114]. TonEBP (or NFAT5, nuclear factor of activated T-cells 5, or OREBP, osmotic response element-binding protein) is a transcription factor modulated by growth factors (GF) [115], cytokines [115,116], and calcium [117]. It is involved also in the survival of NP cells in the hyperosmotic milieu [118]. Together with other osmosensitive pathways and receptors, especially from the mitogen-activated protein kinases (MAPK), transient receptor potential (TRP) channel and Aquaporin family, TonEBP/NFAT5 (tonicity-responsive enhancer binding protein/nuclear factor of activated T-cells 5) plays a crucial role in cell volume regulatory mechanisms [118]. In rat NP cells, extracellular signal-regulated kinase (ERK) phosphorylation following hyperosmotic stress results in TonEBP/NFAT5 activation, thereby promoting cell survival [114,119]. The tight cross talk between ERK and TonEBP/NFAT5 and the link to cell survival/apoptosis have also been demonstrated through pharmacological ERK inhibition [119,120,121]. In addition to MAPK, TonEBP/NFAT5 interconnects to the NF-kB pathway [122,123] and interacts with members of the TRP family [118]. The TRPV subfamily (especially TRPV4) has been identified as potential osmo- and volume-sensors involved in regulatory volume change mechanisms and cell signaling, following osmotically driven opening of the channel pore and subsequent influx of extracellular Ca^2+^ [118]. Consequently, TonEBP/NFAT5 has a wide variety of target genes, ranging from organic osmolytes [124] and aquaporins [125] to ECM molecules [114] and pro-inflammatory cytokines [116]. In particular, aquaporins form transmembrane water channels and are able to regulate intra- and extracellular water balance, which is essential to keep cells alive in fluctuating osmotic environments [125,126,127,128,129].

The IVD is constantly subjected to dynamic loads and the cells embedded within the ECM experience compressive, tensile, and shear mechanical stresses and strains [130]. They respond to these loads via a number of mechanotransduction mechanisms, which have been reviewed previously [130,131,132]. For example, TRP channels, whereby TRPV4 as well as TRPC6, TRPM2, and TRPML1 stand out due to fundamental roles in osmo- and mechano-sensing [118,133,134]. NP cells are more responsive to hydrostatic pressure, while AF cells respond better to cyclic strain [135]. Mechanical loads considered physiological for non-degenerative IVD cells promote matrix synthesis, while higher loading regimes can promote catabolism and contribute to DD [130,131,132]. IVD cells activate distinctive signaling pathways depending on the load magnitude, frequency, and duration, in a zone-specific manner [136,137,138]. Over the past years, the YAP/TAZ signaling has also come into focus in mechanobiology due to its regulation by the mechanical signals elicited by the surrounding ECM [139,140], whereby integrins in focal adhesions (FA) evidently play a crucial role [141]. YAP and TAZ are transcriptional coactivators with involvement in development, tissue homeostasis, tissue renewal/regeneration, and cell proliferation and survival to stress [139]. Previous research clearly indicated that cell stretching over the ECM with reformation of the cytoskeleton causes YAP/TAZ activation, whereas restriction of cell adhesion inhibits YAP/TAZ-related transcription [142]. Such responses were observed when NP cells were cultured in laminin-functionalized polyethylene glycol (PEG) hydrogels with different stiffnesses [143]. In AF cells, the degree of fiber alignment and fiber stress was shown to affect YAP/TAZ activation, with lower nuclear YAP/TAZ in the case of fiber alignment and prestress (highly elongated cell morphology and lower FA area). In contrast, slack and random fibers promoted larger FA and nuclear YAP/TAZ localization [144]. YAP inhibition seems to occur by cell-to-cell contact in IVD cells [145]. Remarkably, while the expression of YAP decreases with age [146], YAP silencing was shown to promote NP cell senescence [145], which adds to the difficulty to duly apprehend the variation of disc cell regulation, upon multifactorial simulation and aging.

IVD cell activity is finely related with careful balance of multifactorial cell cues. Altered balance might result in a vicious cycle of catabolic cell responses and functions [147], which leads to a loss of functional sensitivity to, e.g., mechanical loads at a cellular level, to undue osmolarity, and to ECM depletion over time at a tissue level, which finally results in DD [148,149].

### 3.2. Multifactorial Regulation of Cell Activity in Disease

**Figure 2:**
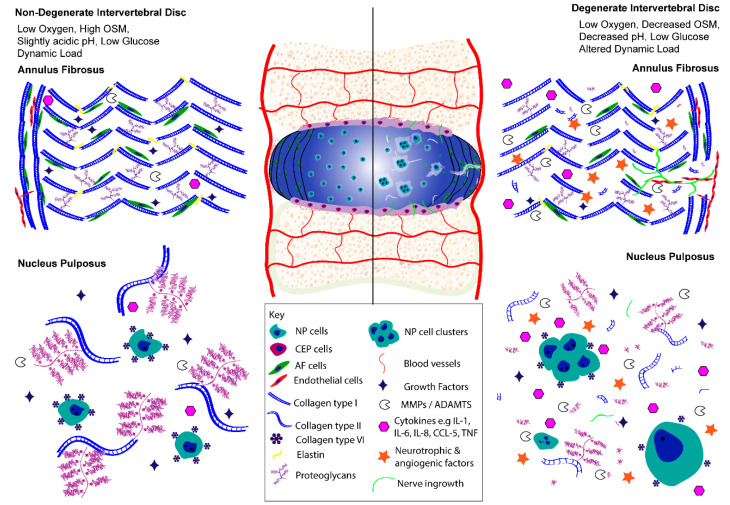
Cellular regulation of the intervertebral disc under degenerated and non-degenerated conditions.

During DD, cellular changes lead to increased production of catabolic cytokines [29,30,150,151,152,153,154,155], matrix-degrading enzymes [4,156,157,158,159,160,161,162], and neurotropic and angiogenic factors [163,164,165,166,167,168,169,170,171,172,173,174,175,176,177], which lead to ECM degradation, catabolism, and nerve and blood vessel ingrowth [178,179,180,181,182,183,184,185,186] (Figure 2).

Furthermore, the number of functional cells decreases with increases in apoptosis, autophagy [187], and cellular senescence [147,188,189,190,191,192,193,194,195]. The initiating trigger of these catabolic events is clearly multifactorial, and different processes are likely to predominate in individual patients, with links to genetics, abnormal loading profiles, infection, and diabetes, among other potential risk factors [196,197,198].

For example, cells from a degenerative IVD respond differently to mechanical stimuli with mechanotransduction pathways altered during degeneration, which further leads to decreased synthesis and increased degradation of the ECM [75,137,199,200,201]. IVD cells respond to altered biomechanics, infection, or metabolic changes with increased production of catabolic cytokines, leading to the generation of a ‘cytokine soup’ regulated predominantly by the pleiotropic cytokine interleukin (IL)-1 [154,202]. The IVD environment becomes progressively more hostile for cells with decreased IVD hydration and nutritional supply, leading to increased lactate production, decrease of pH, and decreased osmolarity [93]. Cells become less able to withstand these conditions and lose the physiological response mechanisms that would maintain homeostasis [116,118,126,203,204]. Low glucose [159,205,206,207] and high lactate concentrations [159,205,207,208] lead to a higher rate of cell death and a catabolic shift in mRNA expression. However, the role of oxygen remains controversial. While it seems that NP cells survive well with limited oxygen levels [159,207], on the one hand, the lack of oxygen has been alternatively linked to either lower [207] or unmodified [209] GAG synthesis by NP or AF cells. On the other hand, a significant rise in aggrecan mRNA expression at 1%, compared to 6% or 21% oxygen concentrations, was found, while mRNA expression for collagen type II was decreased [159]. Other studies report increases in both collagen type II and aggrecan at 1% oxygen [209].

Cell senescence has been reported to be a contributing factor toward the progression of DD, and the causes and molecular mechanisms that are seen to take place were already nicely reviewed [191]. A correlation between age and increased measures of senescence has been shown as well as associations between senescence and elevated MMP and ADAMTS expression [193,210]. As well as losing replicative ability, senescent cells also release pro-inflammatory cytokines and matrix-degrading enzymes. This cell characteristic is referred to as senescence-associated secretory phenotype (SASP) [191]. Secretion of pro-inflammatory cytokines by senescent disc cells includes various catabolic factors, including tumor necrosis factor α (TNF-α) and IL-1β [30].

Apoptosis and autophagy are other important aspects of cell activity in the IVD. The mechanisms of action and roles in matrix homeostasis and degeneration have been reviewed and discussed in detail [187,211,212]. It has been observed in human, animal, and in vitro studies that excessive NP and AF cell apoptosis and autophagy takes place during DD, which may be exacerbated by harsh disc cell microenvironments [187,213]. Autophagy is also important in natural cell and protein turnover within the IVD as low levels have been reported in non-degenerate rat NP and AF cells [214]. However, its role during DD is more complicated, as both higher [215,216] and lower [217] levels of autophagy have been shown. The potentially conflicting roles of autophagy during DD is reviewed and discussed elsewhere [218]. In regards to the role of cytokines, IL-1β has been shown to induce both autophagy and apoptosis in rat AF cells, but only in serum-deprived conditions [219,220], which may be a more reflective condition of the IVD, where nutrient levels are low due to avascularity.

Cell survival and cell death under multifactorial cell environments are strongly controlled though mTOR and Notch cell signaling pathways. mTOR is downstream of PI3/Akt, whereby mTOR is substrate of Akt [221]. Akt can induce direct and indirect activation of mTOR and, similar to PI3/Akt, the protein kinase mTOR has a central role in cell metabolism, growth, proliferation, and survival [222]. Increasing evidence highlights that mTOR controls the decision between cell survival and cell death in case of endoplasmic reticulum (ER) stress [223,224]. In the IVD, mTOR has mostly been investigated in the context of autophagy [187], i.e., an intracellular process that allows cells to remove misfolded or aggregated proteins and eliminate damaged organelles occurring due to stressors such as nutrient deprivation [103,225], oxidative stress [226,227], or overloading [226], thus ensuring cell survival and appropriate cell metabolism [228]. On the one hand, inhibition of mTORC1 promoted rabbit AF and human NP cell survival and reduced catabolic responses under serum and nutrient deprivation as well as by IL-1β treatment via autophagy induction [225,229,230]. Furthermore, the beneficial effect of osteogenic protein 1 treatment on rat NP cell survival under hyperosmotic culture conditions was associated with mTOR (and PI3/Akt) activation [231]. Interestingly, mTOR inhibition has also been found to affect matrix synthesis and degradation in the IVD in mice, with reduced aggrecanolysis (likely via reduction in cell senescence) but simultaneous suppression of PG synthesis, thus not leading to any changes in total PG content [232].

The Notch signaling pathway is a highly conserved pathway with a wide variety of functions in development, tissue homeostasis and diseases, ranging from stimulation of tissue growth to promotion of cell death under different cell microenvironments [233]. As transmembrane receptors with a direct route from the membrane to the nucleus, Notch 1-4 can only exhibit such diverse functionality by a range of regulatory mechanisms such as tissue topology, ligand expression patterns, expression of certain enzymes, or the extent of cell–cell contact [234]. In IVD cells, the expression of Notch 2 is increased during DD [235], whereas intradiscal injection of JAG2 (which induces Notch 2) reduced DD processes in rats [236]. Notch signaling in the IVD was activated by hypoxia [237] and pro-inflammatory cytokine exposure [235], thereby activating NP and AF cell proliferation [236,237], inhibiting NP cell apoptosis promoted by TNF-α [236] and modulating the expression of anabolic and catabolic genes [238]. Yet, these effects seem to be zone-dependent, with Notch activation causing catabolic and anabolic responses in AF and NP cells, respectively [238]. Importantly, cross talk between the Notch signaling pathway and MAPK, NF-kB, PI3K/Akt, and Wnt/β-catenin (Section 4.1) seems to exist [235,236]. Despite these fascinating findings, relatively little research has thus far been conducted on the Notch pathway in the IVD.

## 4. IVD Regeneration Strategies: Biological Targets and Biomaterials

Given the high incidence of DD and the related high financial burden [47] and global disability [239], IVD regeneration is a crucial focus in IVD research. Apart from conservative approaches, regeneration due to a manipulation of biological targets, cell therapy-based strategies, biomaterials, and nanotechnologies to, for example assimilate the architecture of biomaterials to the native tissue or to optimize drug delivery, is currently under investigation. The following subsections provide an overview of the current hopes in IVD regeneration strategies.

### 4.1. Signaling Pathways and Biological Targets

As illustrated in the previous sections, the multifactorial regulation of IVD cell activity by cues such as mechanical loading, osmolarity, glucose, hypoxia, and paracrine and autocrine factors results in complex cell signaling processes that are often interconnected. Understanding the regulation and interconnectivity of various pathways is crucial for elucidating the complex mechanisms of DD, especially when the goal is to develop novel molecular treatment options.

Over the past years, the IVD research community has identified numerous promising biological targets. Modulating these targets, e.g., pharmacologically or through genome engineering approaches such as CRISPR/Cas [240], may allow interfering with the molecular and biological mechanisms of DD and/or pain development. Consequently, the hope is to develop more effective and less invasive treatment options compared to currently available strategies in patient care. Some of the most promising therapeutic targets are, for example, MAPK, NF-κB, Wnt/β-Catenin, and PI3/Akt.

MAPK are a family of highly conserved signal transduction pathways that facilitate mammalian cell responses to numerous extracellular signals. MAPK activation occurs as a cascade, whereby each member of the family of MAPK is activated by specific upstream kinases (MAPKK), which are in turn activated by a MAPKK kinase (MAPKKK), all by phosphorylation [185]. In mammals, three major subfamilies of MAPK exist: ERK, the c-Jun NH2 terminal kinases (JNK), and the p38 isoforms (p38 MAPK) [186,187]. MAPK can be activated by numerous cell stimuli present in the IVD and have thus been discussed as potential therapeutic targets in DD [241].

- ERK activation typically occurs via mitogens and GF (e.g., platelet-derived growth factor (PDGF), transforming growth factors β1 and β3 (TGF-β1, TGF-β3), fibroblast growth factor (FGF), and insulin-like growth factor (IGF) I [242,243,244]), thereby controlling growth, differentiation, cell cycle progression, and development. In addition, ERK activation in the IVD supports cell survival following hypoxia and osmotic stress, the latter with cross talk to TonEBP [118,119,120,121,245,246,247]. Interestingly, NP-derived mesenchymal stromal cells (MSC) also respond to osmotic stimuli, whereby hyperosmotic stress was associated with ERK activation, leading to a reduction in proliferation and chondrogenic differentiation [248]. Interestingly, excessive cyclic stretch was shown to induce AF apoptosis via inhibition of ERK phosphorylation, whereby β1 integrin could inhibit the apoptotic processes [249]. Pro-inflammatory cytokines, such as TNF-α and IL-1β, as well as stimuli known to induce inflammation, such as ECM fragments, activate the ERK pathway in IVD cells, possibly mediating loss of tissue ECM proteins associated with DD [31,55,250,251,252,253], inflammatory and catabolic responses [250,254,255], apoptosis [256], and senescence [257]. Interestingly, ERK was suppressed by stimulation with the anti-inflammatory cytokine IL-10 [254]. Overall, these findings indicate that modulating ERK activity for therapeutic means is possible yet challenging due to the multifactorial role of this signaling pathway.
- The p38 signaling pathway is generally activated by stressors and is known to regulate inflammation, autophagy, apoptosis, and differentiation [241]. Numerous studies have investigated p38 in the IVD, thereby identifying hypoxia [245], hyperosmolarity [120], hyperphysiological mechanical loads [133], ER stress [258], acidity [257], high glucose levels [256], and IL-1 [253] as potent activators. Interestingly, p38 is connected to TPRV4 [133], which has previously been described to transduce mechanical, inflammatory, and pain signals in cartilage [259]. Different research fields have shown extensive cross talk between p38 and other signaling pathways, e.g., ERK [258], TGF-β/Smad, [260] or Akt [261], which should be investigated in IVD cells. Overall, inhibition of p38 is being discussed for therapeutic approaches, potentially reducing inflammation, pain, and disc matrix catabolism [253,262], although ultimate outcomes may be difficult to predict due to the extensive cross talk with other pathways.
- JNK, similar to p38, is activated by stressors, GF, and pro-inflammatory cytokines [241,253]. Stressors entail high glucose levels [256], hyperosmolarity [120,263], TNF-α and IL-1β exposure [250,251,255], syndecan-4 overexpression [264], and Propionibacterium acnes (P. acnes) infection [265]. Following activation, JNK regulates apoptosis [120,256,265], enhanced expression of MMP [250], DNA damage [263], and DD [264]. The pro-apoptotic mechanisms of JNK seem to be associated with p53 induction [266] and with toll-like receptor 2 activation [265]. Although not yet investigated in the IVD, the interaction of JNK with miRNAs (e.g., miR-138, miR-133a-3p, miR-133b-3p, miR-4268) is likely relevant [267,268,269]. Therefore, a better understanding of JNK signaling will be needed before its modulation can be effectively used as a therapeutic means.

NF-κB is described as the master regulator of inflammation. As it has become increasingly clear that aberrant regulation of the NF-κB signaling pathway intervenes in DD, studies have started to investigate its potential clinical use of NF-κB-targeting therapies [270]. This signaling pathway is activated in response to damage, pathogens, and cellular and mechanical stress and inflammation and regulates the expression of numerous genes related to inflammation, catabolism, and apoptosis/cell survival [241,271]. In mammals, it consists of a family of dimer-forming transcription factors that share the Rel homology, namely RelA (p65), c-Rel, RelB, p50, and p52 [272]. In the IVD, p65 expression and more importantly NF-κB activation are increased with degeneration [253,273], and pro-inflammatory cytokines, such as IL-1β and TNF-α, shown to activate NF-κB [241,250,253,273,274], are associated with painful DD [153,275]. Downstream targets of NF-κB include numerous matrix-degrading enzymes (*MMP-1/-2/-3/-13, ADAMTS-4/-5*), ECM proteins (*asporin*), inflammatory mediators (*iNOS, COX-2, prostaglandin E*), and chemokines (*MCP-1*), supporting the crucial role of this pathway in IVD and inflammation and degeneration [153,250,274,275,276,277,278,279]. In addition to specific NF-κB inhibitors such as ACHP (2-amino-6-[2-(cyclopropylmethoxy)-6-hydroxyphenyl]-4-(4-piperidinyl)-3-pyridinecarbonitrile) or the NF-κB essential modulator (NEMO) binding domain peptide (NBD) [275,280], several natural drugs (e.g., curcumin [281], epigallocatechin gallate [255], or resveratrol [282]) have been described to modulate NF-κB activity. Furthermore, NF-κB signaling can be altered by specific miRNAs (e.g., miR-150 [283]), but NF-κB activation can also affect expression of numerous miRNAs (e.g., miR-640 [284]). These findings offer new therapeutic approaches as miRNAs play a crucial relevance in IVD ECM degradation, cell apoptosis, and inflammation, e.g., due to their role as post-transcriptional regulators [285].

Wnt/β-Catenin represents another therapeutic target in DD. It is a highly conserved pathway, involved in cell fate decisions during development, also regulating cell proliferation and tissue growth and maintenance [286]. Interestingly, Wnt signals are often only effective in localized areas between neighboring cells [287]. Activation of the Wnt/β-Catenin pathway is initiated by binding of Wnt proteins to the so-called Frizzled receptors on the cell surface, which allows the transcriptional co-regulator β-catenin to shuttle to the nucleus, where it activates transcription of Wnt target genes [286,287]. Through mouse models, Wnt signal activity could be demonstrated during IVD development in the AF and the CEP [288], as well as early in life in the NP (likely associated with the postnatal rapid growth phase). In contrast, it was found to be downregulated with age [289] and DD [290,291], possibly through miR-532 [292]. Thus, activation of Wnt/β-Catenin may have the potential to reverse age-related degenerative changes in the IVD [289,290]. In fact, overexpression of Wnt in human herniated NP cells increased GAG release [293], supporting the possible therapeutic potential of the Wnt/β-Catenin pathway. However, Wnt/β-Catenin signaling was seen to induce senescence in IVD cells while also interacting with TNF-α in a positive-feedback mechanism, potentially contributing to disease progression [210,294]. Therefore, activation of Wnt/β-Catenin will only become therapeutically relevant when possible unwanted side effects are identified and controlled.

As illustrated in Section 3.2, PI3/Akt is a well-known cell survival pathway and also regulates metabolism, proliferation, cell cycle progression, growth, and angiogenesis [295]. Due to its multifactorial role, PI3/Akt is tightly controlled, e.g., by its inhibitors as well as by cross talk with NF-kB [296,297,298]. The PI3/Akt pathway is primarily activated by cytokines [296] and GF [299,300,301] and is a critical player in DD [302]. GF activation, for example, resulted in Akt-dependent aggrecan accumulation in bovine NP cells [303] as well as reduced autophagy [302]. Akt has also been shown to positively regulate cell proliferation [243] and counteract DD processes [300]. Natural drugs, such as epigallocatechin gallate and resveratrol, can activate PI3/Akt under stress conditions, thus stimulating important and therapeutically promising pro-survival mechanisms in IVD cells [304,305]. In line with that, inhibition of miR-4458 or miR-27a or stimulation of miR-21 may also have therapeutic benefits through PI3K/Akt modulation [306,307,308].

In addition, membrane receptors known to play a role in multifactorial disc cell regulation (see Section 3.1) are being investigated as therapeutic targets in DD. On the one hand, TRP channels have emerged as drug targets [134], due to correlations with pain intensity and duration [309]. On the other hand, toll-like receptors clearly hold promise for the treatment of DD [164,310,311,312]. They are tightly associated with MAPK and NF-κB signaling and, thus, with inflammation and catabolism. One of the downstream targets of toll-like receptor 2 is NGF (nerve growth factor) [164], which plays a crucial role in IVD innervation and pain development. Thus, anti-NGF therapeutics may have the ability to manage pain in DD [167,185,313]. In this context, Link N, a naturally occurring peptide, could be of clinical relevance thanks to its inhibitory effect on NGF expression and to its regenerative capacity [314].

### 4.2. Growth Factor-Based Strategies

A variety of GF have been studied for their capacity to encourage IVD regeneration, including PDGF, IGF-1, and FGF18, although the majority of GF that have been studied belong to the TGF superfamily [315]. Members of the bone morphogenetic protein (BMP) (mainly BMP2, BMP7) family and of the TGFβ (TGFβ1 and TGFβ3) subfamilies have been studied extensively, using in vivo models, to encourage anabolic activity in resident NP cells and counteract pathology [315,316]. In vivo testing of BMP has been mixed, with positive results shown for BMP2 and BMP7 in a small animal (rabbit) but negative results in a large animal (goat and canine) models [317,318]. More encouraging and more recent data have come from members of the growth differentiation factor (GDF) family (also part of the BMP family). GDF has been shown to have anabolic effects on IVD cells with GDF6 (BMP13) also having potent chemoattractant properties for NP cells [319]. In vivo testing of GDF6 has shown positive effects in sheep, rat, and rabbit models with decreased signatures of degeneration and evidence of disc tissue restoration [320,321]. GDF5 (BMP-14), due to promising preclinical studies [322], has been tested in two phase I/II clinical trials that are now complete [323]. Unfortunately, the placebo-controlled phase II trial failed to show efficacy; however, the patient sample size was small (N = 45). Although preliminary results of this type of potential treatment are promising, there are still challenges facing GF-based therapies. Firstly, they are inherently reliant upon the remaining degenerated disc cells to be healthy and sufficient in number to synthesize ECM and ultimately regenerate the disc tissue. Secondly, GF are often short-lived, limiting clinical use toward sustained regeneration [324]; hence, recent works were investigating the use of microparticles as a GF delivery vehicle [325,326,327]. Arguably, this is a likely contributing factor to the lack of efficacy from the GDF5 clinical trial. Cell replacement therapy, on the other hand, circumvents both of these challenges.

### 4.3. Cell Therapy-Based Strategies

A wide range of potential cell-based therapies has been proposed for IVD regenerative strategies, and there have been extensive reviews on the use of cellular therapies for regeneration of the IVD [328,329,330,331]. Cellular strategies have ranged from studies on terminally differentiated chondrocytes [332,333,334] to more tissue-specific cell sources such as native disc cells from mature NP tissues [335,336,337] or immature notochordal cells from porcine discs [338,339]. There have been extensive studies using a number of stem cell sources, including MSC from bone marrow or adipose tissues [340]. More recent studies are exploring the potential of induced pluripotent stem cells in the treatment of DD [341,342,343,344,345,346,347], and there are a number of ongoing clinical trials that use cellular injections into degenerated IVD [348,349,350,351]. A number of recent reviews have been published proposing recommended routes to develop these therapies [93,352], which highlight important considerations, particularly focused on the potential fate of cells injected into the harsh environment of the degenerated discs [352,353,354,355,356,357]. The path to successful therapies is likely to combine cellular therapies with molecular targets (see Section 4.1), to inhibit the degenerated niche, together with biomaterial strategies to provide proper support for the cells during delivery.

### 4.4. Biomaterials and Nanotechnologies

Biomaterials serve a fundamental role in tissue engineering (TE) by acting as scaffolds for in situ tissue replenishment as well as being carriers for cells and biological molecules. A number of review articles provide summaries of the numerous and varied biomaterials suitable for the IVD [358,359,360]. Often, the biomechanics of the native tissue is replicated as closely as possible with the design of the biomaterial, along with the ability to encourage adherence, growth, and/or differentiation of cells. Since the IVD is composed of interconnected regions, each with different physical properties, the appropriate biomaterial for one region is generally different to the other. The NP demands most TE strategies to employ the use of hydrogels as they are hydrophilic [361]. Conversely, biomaterials’ research for AF TE targets rather fibrous organized constructs, often achieved through electrospinning [362,363]. However, the natural complexity of the AF structure and composition is uniquely designed to resist an amazing variety of mechanical loads, both in nature and magnitude, which makes AF TE particularly challenging [363,364]. Indeed, integrated replacement strategies for the whole IVD are also commonly explored targeting both the NP and the AF, through highly hydrated composites [365,366,367]. Evidence has been predominantly collected in vitro/ex vivo, whereas in vivo studies comprise a far smaller proportion, which relates to the need for development of appropriate animal models previous to clinical trials. Remarkably, appropriate animal models to test disc regeneration strategies shall be conditioned by the existence of corresponding models for DD, which remains a challenge *per se* [368]. Evidence of this shortcoming comes from a 2014 meta-analysis of in vivo and clinical studies for DD that reported only four clinical studies that utilized biomaterials [369]. Hence, continued progress is required in the field of IVD TE to increase translational research and the number of clinically approved biomaterial options for patients. Table 1 summarizes both key biomaterials and the related evidence that have been achieved for disc TE.

Microparticles (MP) are another type of biomaterial employed in IVD TE strategies. MP have long been explored within the pharmaceutical industry for use in drug delivery [437]. By tailoring their design, controlled temporal and dose release of biologicals can be achieved, e.g., to reach the biological targets reviewed in Section 4.1. Furthermore, controlled administration of biologicals is extremely valuable for IVD TE, where cell and/or growth factor therapeutics would rely on single surgical procedures rather than on repeated delivery. In vitro, delivery of GDF6 through MP was demonstrated to be more efficient than repeated exogenous delivery to differentiate adipose stem cells into NP cells [327], which provides a promising direction for single surgical procedure approaches. MP have also been used to release the anti-inflammatory factor cyclooxygenase-2 in a canine dog model of DD [438], leading to encouraging results of preventing disease progression and reducing the inflammatory signature prostaglandin E_2_, a crucial nociception mediator.

More recently, the advancements of nanomaterial technologies have lent themselves to IVD applications [439], allowing the tissue architecture to be replicated at a much finer scale than before, hopefully leading to biomaterials eventually indistinguishable from native tissue. Examples include poly L-lactic acid combined with nanofibrous scaffold to mimic the AF [430] and PGA-chitosan nanocomplex for NP regeneration [440]. Furthermore, nanomaterials are also being applied for growth factor delivery, such as assembling nanoparticles loaded with bFGF (basic fibroblast growth factor) onto microspheres for discogenic differentiation of MSC [441]. There is no doubt that the ever-advancing field of biomaterials will play a key role in providing solutions for DD in the future, whether that is supporting cell/drug delivery or acting purely as structural support for innate tissue regeneration.

## 5. Systems’ Modeling for the Exploration of IVD Degenerative and Regenerative Mechanisms

**Figure 3:**
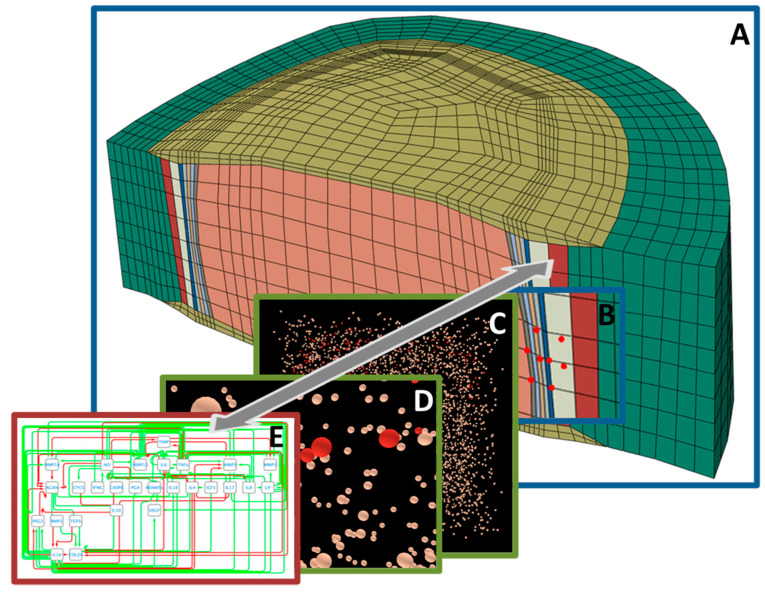
In silico multiscale/integrative modeling as an objective in IVD research. (**A**) Organ level: 3D finite element modelling. Deformable nonlinear geometry, heterogeneous assembly of tissues. (**B**) Tissue level: 3D finite element modelling. Composition-dependent multiphysics and anisotropic behavior of the tissues, transport of solutes through tissue matrices. (**C**,**D**) Multicellular levels: agent-based modeling in regions of interest. Prediction of cell responses to multifactorial (micro-) environments. (**E**) Cell/subcellular levels: network modeling. Single cell stimulation, multiple states.

Successful IVD treatment/regeneration strategies rely on a holistic understanding of the highly multifactorial (patho)physiological dynamics of the disc system, to be understood at different time and length scales. In this sense, theoretical and computer modeling offers unique possibilities (Figure 3). 

For musculoskeletal joints commonly affected by highly prevalent disorders such as osteoarthritis, efforts in model developments have spanned over the scales, from the multibody musculoskeletal system to cell regulation networks, passing through detailed knee joint finite element models [442]. Even if the systematic integration of models of different nature at different scales is still incomplete, developments use to be much more modest as far as the IVD and DD are concerned. Indeed, model developments to simulate and virtually explore the pathophysiology of DD have long remained largely limited to the tissue and organ scales [443]. Only very recently, cell-scale models have finally emerged out of novel integrations of knowledge in IVD cell biology and computer methods in systems biology [35]. Arguably, the holistic modeling of the heterogenous IVD system and the degeneration thereof is a tremendous challenge that will surely contribute to further progress in the development of in silico tools and medicine in other fields of rheumatology.

On the one hand, the process of model construction and assessment against evidences provides important clues about the minimum hypotheses and quantitative factors essential to reproduce known phenomena in health and disease. On the other hand, the huge capacity for parametrical studies turns in silico models into unique virtual laboratories to design new hypotheses and experiments. Eventually, model predictability, even in terms of relative analyses of simulation results, i.e., semiquantitative predictability, is definitively cornerstone to enable mechanistic explicability and better control of patient stratification and treatment prognosis. Significant progress in IVD modeling has been made during the last years at multiple scales and is summarized below, along with relevant findings about IVD regulation, DD, and possible therapies.

### 5.1. Organ- and Tissue-Scale Simulations of the IVD Biophysical Regulation

IVD tissues can be considered as biphasic materials, i.e., a combination of solid and liquid phases, at the millimetric scale. The porous solid phase corresponds to the tissue ECM, i.e., mostly collagen and PG. The liquid phase is mainly composed of water and solutes that flow through the pores of the solid phase. Biphasic mixture theory and Biot poroelastic theory allow to model IVD tissues and discriminate the respective roles of the solid and the fluid [444,445], especially in terms of mechanically coupled solute transport to the cells, for further simulations of cell activity [443]. The effect of mechanically coupled solute transport on cell activity has been referred to as indirect mechanotransduction, which depends on the capacity of the IVD tissues to deform [132] and control both the diffusion distances and the porosity, i.e., water content, in the disc [446]. Obviously, the whole process depends on tissue composition, i.e., on the effective condition of the IVD.

Composition-based tissue models were developed for the articular cartilage [447] and were later applied to the IVD [448]. Implemented into detailed finite element models of the whole IVD, these models included the osmotic pressure in the disc tissues, controlled by PG, collagen, and water contents as explicit model parameters. Strain energy density calculations in the AF further considered the anisotropy induced by the oriented collagen type I. Composition-dependent tissue modeling has paved the way to map the relative effects of local ECM depletion, in the NP, the AF, and the CEP, on the biphasic behavior of the IVD and on the indirect mechanotransduction phenomena [37,68]. Such tissue models were also coupled to phenomenological direct mechanotransduction theories, initially developed to predict the fate of bone MSC in endochondral bone healing, to calculate the likely long-term IVD remodeling after spine surgery [449]. The effect of PG and fixed-charge density contents on the diffusion of antibiotics from the vasculature to the IVD was also assessed through finite element mechano-transport simulations [450,451].

The hypothesis that impaired diffusion of metabolites across the IVD might accelerate DD through cell nutritional stress [2,34] has motivated experimental measurements of cell viability in function of local pH and glucose concentrations in a diffusion chamber [207] and the establishment of empirical relationships between the oxygen, glucose, and lactate metabolism by IVD cells [98]. These works have been instrumental for the implementation of the aforementioned mechano-transport models, to couple IVD tissue mechanics, IVD morphology, local cell metabolism, and cell death [443]. Such simulations revealed that water contents in the disc, especially in the NP, largely control the effective diffusion of solutes, as well as the diffusion distances, under physiological mechanical loads [446,452].

The diffusion process in the disc ECM is relatively low and disc cell populations can withstand adverse nutritional environments during several hours to a few days before dying [207]. Accordingly, model simulations point out that the effect of molecule mechano-transport on IVD cell activity becomes remarkable under sustained rather than under transient mechanical loads [446]. Furthermore, mechano-transport simulations suggested that indirect mechanotransduction on the long term, i.e., decades, might explain natural aging in the IVD [36]. Interestingly, a collection of patient-specific IVD models showed that large lumbar discs, i.e., higher than 14 mm, might be prone to spontaneous degeneration in contrast to average seize IVD, i.e., 8–12 mm high, because diffusion distances were too large to allow the nutrients to reach the cells in the center of the NP [453].

Remarkably, models and simulations have proven great ability to identify specific risk factors and multifactorial mechanisms. Endplate obstruction because of sclerosis/calcification has been long suspected to be responsible for nutritional disturbances in the IVD [454,455,456]. However, micro-modeling of vertebral endplate specimens coupled with full IVD finite element simulations suggests that the variability of the calcified endplate structure and porosity with aging and degeneration is unlikely to generate any barrier able induce nutritional stress in the IVD [457]. In contrast, the use of a composition-based IVD model showed that the early depletion of CEP in terms of PG and collagen type II increases the overall permeability of the endplate, with a specially high influence of the loss of PG [37]. Such a permeability increase was also measured with aging [59], and simulations indicated that it might provoke a chronic dehydration of the NP, down to water contents characteristic of Pfirrmann grade III degenerated discs, under daily physiological loads. This reduction of water largely reduced the capacity of the nutrients to reach NP cells located in the anterior part of the IVD, close to the AF. Interestingly, these results may provide mechanistic explanations of the severity of endplate-driven DD and LBP, as eventually revealed by Modic changes and vertebral endplate defects, in the general population [21,458,459]. Yet, such level of modeling cannot explain the pathophysiology of the CEP degeneration, per se, for which it is necessary to go down the scale.

### 5.2. IVD Cell Models and Integration of Experimental Cell Stimulation Data

As reflected through Section 3 and Section 4, experimental studies at a cellular level increased our understanding of anabolic or catabolic processes by IVD cells, and biochemical, metabolic, and mechanical stimuli that affect cell activity could be revealed. However, critical interactions within the multifactorial environment to which a cell is exposed over long periods of time are difficult to capture experimentally or clinically, pointing out the need for in silico approaches down to the cell level.

Recently, a first agent-based modeling approach was proposed to simulate the behavior of NP cells in multicellular systems depending on biochemical microenvironments [35]. The model uses experimental findings to estimate cell viability and cell activity in terms of relative mRNA expressions of *collagen types I* and *II* and *aggrecan* and of *MMP* and *ADAMTS* proteins, based on user-defined nutritional factors and on inflammation. It exploited network modeling approaches from systems biology [460], to integrate the respective effect of different micro-environmental cell cues on effective cell activities. It was then further developed to integrate direct mechanotransduction, i.e., load magnitude and frequency, effects [461]. On the one hand, such an approach informed about the likely differential behavior of non-inflamed and inflamed NP cells in similar microenvironments. On the other hand, simulations captured the expected relative influence of different mechanical load regimes on the capacity of NP cells to retain a full anabolic activity or provoke ECM depletion.

Complex interactions at a (sub)cellular level can be approached through different modeling formalisms such as Boolean or Bayesian networks, Petri nets, constraint-based, rule-based, or agent-based models, differential equations, process algebra, interacting state machines, or cellular automata [462]. Still incipient in IVD research, models in systems biology often focus on one type of network, mainly metabolic-, signaling- and gene-regulatory networks, facing limitations in interconnecting these networks toward holistic representations of cell simulations [462]. Future developments shall couple systems biology approaches and organ/tissue-scale finite element models (Section 5.1). As such, the multiscale dynamics that control the IVD fate, and the mechanisms that lead to different phenotypes of IVD failure, will be represented, thereby allowing the mechanistic identification of new therapeutical targets. Furthermore, integrating cell regulation pathways into single and collective cell behavior models could uniquely bridge the gap between IVD tissue phenotypes and the intracellular machinery to explain the apparent links with different genetic variants [57,463].

### 5.3. Cell Signalling Pathway Models and Integration of Multi-Omics Data

As exposed in Section 3, cell signaling (or signal transduction) is the process that describes how cells communicate with their environment and how they respond to external or internal stimuli [464]. Signal transduction pathways describe the transformation of a stimulus into a biochemical signal often starting with ligand-receptor binding, followed by an intracellular cascade of protein–protein interactions and resulting in a cellular response/cell fate decision such as expression of a certain gene, apoptosis, proliferation, etc. Signal transduction plays a fundamental role in cellular behavior and the high complexity of cell signaling has led to many mathematical modeling approaches in order to better understand the underlying dynamics and deduce quantifiable conclusions [465]. In general, signaling pathways can be described as node-edge graphs (directed or undirected) with the proteins as the nodes and the edges as the interactions between the proteins. These protein–protein interaction networks are stored in several publicly available databases and aim to represent the existing knowledge of the scientific literature about either the entire interactome or the structure of specific pathways. Some of the popular databases are presented in Table 2, which can serve as a starting point for the creation of signal transduction pathways.

After extraction of the pathways of interest, prior-knowledge network (PKN) can be modeled with various mathematical approaches. The most widely used ones are either logic modeling employing Boolean or fuzzy-logic formalisms [473,474,475] or ordinary differential equation models [476,477]. Whereas the former ignores time-dependent behavior and assumes instantaneous state changes of the system, the latter can describe transient behavior at a cost of a high number of reaction parameters that are difficult to identify experimentally. Interestingly, methodologies as presented by Mendoza and Xenarios [460] and Krumsiek et al. [478] use a generalized formulation to transfer discrete node-edge graphs into continuous systems, to include dynamics into logic models.

Although considerable effort has been put to create databases on protein interaction and cell signaling pathways, the existing information has and will always have limitations, e.g., biases (well-known proteins are studied more often than under-reported players), assumptions during the data mining algorithms, and ambivalent behavior of individual protein pairs (stimulation and inhibition). The best approach to overcome this intrinsic limitation is to fit the PKN structure to our own experimental data. On this front, various massive parallel sequencing platforms can be used to provide systematic molecular profiling of human cells, which will be tackled in the next few years by the European Innovative Training Network Disc4All in IVD research [479]. The technologies are summarized under the term ‘*omics*’, considering, e.g., genomics, proteomics, metabolomics, lipidomics, and other high-throughput technologies [480].

An attractive techniques allowing protein detection with high sensitivity and specificity as well as relatively simple experimental protocols are multiplexed ELISA immunoassays on magnetic beads [481]. This technique allows the simultaneous measurement of phosphoprotein activity and cytokine abundance (several dozens in one sample) after stimulation of biological samples [482]. This high-dimensional data serves as a starting point to optimize and fit the developed signaling pathway models. Here, either genetic algorithms [483] or integer linear programming formulations [484] are viable approaches to systematically fit node-edge graphs to the experimental data. In particular, the CellNOpt [485] and SigNetTrainer that is part of the CellNetAnalyzer toolbox [486] offer user-friendly ways to perform such optimization routines. A recently developed alternative is DoRoTHea [487] that creates signal transduction graphs based on gene expression measurements taken, for example, from microarray studies. In the end, these pathways models can be used to investigate the overall dynamics of the system, identify the most influential nodes, or focus on the specific parts of the signal transduction network to see the effect of various stimuli and/or knockouts [465].

Many pathways are involved in IVD cell regulation [187] and their individual analysis has been performed in the past in a vast amount of studies [488,489,490,491]. Until now a holistic representation of pathways in the IVD is still missing and a possible approach to tackle this obstacle might follow the strategy described above, as proposed in the Disc4All project [479]. Especially, the requirement of high-quality experimental data (ideally from relevant in vitro models) is one of the major challenges, in order to capture the mechanisms of multifactorial diseases such as DD. However, the falling costs of high-throughput technologies will lead to increasing amounts of publicly available molecular information. The rapid developments in computational biology tools might then bridge the gap between information and mechanistic knowledge in DD and IVD regenerative therapies.

## 6. Conclusions

This review provides an integrative view of the current knowledge about the non-degenerated and degenerated IVD at the tissue, cell, and molecular levels and of current disc regeneration strategies. It further summarizes recent modeling approaches that illustrate the capacity of in silico models to simulate complex interactions over different time and length scales and supports improved identification of risk factors and the effect thereof at the molecular level, for improved targeted therapies.

At the tissue level, sophisticated interactions among PG, water, and different types of collagen are crucial to keep the IVD mechanically competent, also ensuring proper cues at the cell level for optimal tissue maintenance, regardless of the harsh mechanical, osmolar, metabolic, and pH environments. Despite the highly elaborated responses of IVD cells to their adverse microenvironment, IVD homeostasis is fragile, and multifactorial perturbations of cellular cues might trigger a cascade of catabolic responses, such as increased synthesis of catabolic cytokines, matrix-degrading enzymes, or neurotropic and angiogenic factors, which gradually affects ECM integrity.

Those responses are triggered by complex and interconnected cell signaling pathways, the detail of which becomes better known, based on multiple controlled experiments that exploit gene-editing techniques, among others. Such understanding is of utmost importance to develop new molecular treatments and is being exploited in regenerative strategies based on pharmacological solutions. The challenge resides, however, in the proper anticipation and control of the differential expression of the pathways and cross talks, when the IVD cells are subjected to complex cocktails of stimuli, part of which depend on top-down mechanical, nutritional, and chemical spatiotemporal events.

Cellular therapies for IVD regeneration have been widely studied with several potential cell sources investigated. However, given the harsh conditions within a degenerated IVD and the need to control mechanotransduction effects, combined cell therapy, molecular targets, and biomaterials approaches shall be the most promising approaches. Because of the complexity to thoroughly apprehend the effect of such combinations, holistic understanding of top-down and bottom-up propagations of the consequences of (non-)altered cell activities shall be necessary.

In silico modeling approaches were shown to have great potential to simulate multifactorial mechanisms and identify the effect, down to the cell level, of specific risk factors across the scales. These approaches are still relatively recent and even incipient (e.g., at the molecular and cell scales) in IVD research. Yet, ongoing studies are providing robust proofs of concepts of the exploitability of computer models and simulations and open new horizons in IVD science, by simulating multicellular systems through network- and agent-based modeling. The control of these calculations from the tissue and organ scales can be described through multiphysics and finite element modeling. Latest explorations in this sense targeted mechanotransport and indirect mechanotransduction phenomena and results pointed out the importance of different spatio-temporal events that involve the entire IVD. Eventually, models and simulations can stand for virtual labs, thereby allowing us to virtually test numerous regeneration approaches that will be difficult to anticipate or extremely costly otherwise.

## Figures and Tables

**Table 1:** The major natural and synthetic biomaterials that have been studied for their potential use for treating intervertebral disc degeneration (DD). In the context of intervertebral disc (IVD) applications, potential strengths (+) and drawbacks (−) for each material are provided as well as the IVD-specific studies accompanying them.

Hydrogels for IVD TE		
Natural Hydrogels		
Type	Material Biomechanical Properties	IVD Studies
Alginate	+ Injectable, biocompatible, tailorable properties, anionic properties attract cationic PG [358]. − Diminishing structural integrity over time in calcium cross-linked hydrogels [370].	In vitro/ex vivo: In situ gelation of calcium carbonate cross-linked alginate hydrogel showing ability to maintain disc height over cyclic loading regime [371]. In vitro: Porcine AF, NP, and transition zone cells were cultured in sodium chloride cross-linked alginate beads demonstrating IVD relevant ECM expression but diminished mechanical properties [370]. In vitro: Bovine NP cells encapsulated in a photo-cross-linkable alginate hydrogel showed decrease cell viability over 14-d culture period [372].
Fibrin	+ Biocompatible, gelation time control, biodegradable, promotes matrix synthesis stem cell-derived chondrocytes, non-immunogenic [366]. − Soft in nature (however, can be modified to overcome this) [373].	In vitro/ex vivo: Fibrin-genipin hydrogel with silk scaffold for AF and NP repair demonstrated cytotoxicity to cells in vitro and no recovery of disc height but matrix comparable to healthy disc in bovine organ culture [374]. Ex vivo/in vivo: Fibrin-genipin adhesive hydrogel tested in bovine organ culture and in a mouse model for AF defect repair demonstrating biocompatibility and biomechanics’ restoration [375]. Clinical: A phase II, randomized, double-blind, placebo-controlled study. Assessment of safety and preliminary efficacy of juvenile chondrocytes delivered using a fibrin carrier (NuQu^®^) for treating disc pain [376].
Collagen	+ Good cell adhesion, biocompatibility, and proliferation. Non-immunogenic. Major component of IVD. − Poor mechanical properties with high degradation rate [361].	In vitro: Dense collagen I hydrogel demonstrating comparable functional characteristics to NP [377]. Ex vivo: Condensed collagen gel for NP replacement showed disc height restoration but extrusions of implant during stress testing [378]. Composites: In vitro: Human NP cells encapsulated type II collagen-hyaluronic acid hydrogel crosslinked 1-ethyl-3 (3-dimethyl aminopropyl) carbodiimide demonstrating cell proliferation but no increase in matrix gene expression compared with control gel [379]. In vivo: Transplantation of HA/collagen hydrogel into porcine nucleotomy model causing localized annular damage and inflammation [380].
Atelo-collagen	+ Low immunoreactivity, injectable due to liquid–solid transition when warmed to body temperature, support high-density cell cultures [360].	Ex vivo: Autologous MSC encapsulated in atelocollagen II gel and transplanted into IVD of rabbit disc degeneration model resulting in disc height recovery and PG accumulation [381]. In vivo: Autologous MSC encapsulated in atelocollagen II gel and injected into degeneration-induced NP of rabbits resulting in comparable PG accumulation to healthy control [382].
Chitosan	+ Supports IVD cell encapsulation, cationic properties retain PG, thermoresponsive [383]. − Cell adhesion and mechanical properties not ideal for IVD [358].	In vitro*:* Bovine IVD cells encapsulated in chitosan hydrogel showed retention of NP-produced PG within gel. Gel cytotoxic towards AF cells [383]. In vitro*:* Human MSC differentiation into NP-like cells in a chitosan-glycerophosphate hydrogel [384].
Gellan gum	+ Thermo-reversible gel properties, acid and heat resistant, non-cytotoxic, gelation without the need of harsh reagents, supports chondrocyte ECM deposition [360,385]. − Mechanically weak, requires high gelling temperatures, and lacks anchorage sites for adherent dependent cells [386].	In vitro: Ionic and photo-cross-linked methacrylated gellan gum showed lower water uptake ability but improved mechanical properties than gellan gum alone in the context of NP repair [385]. In vitro/In vivo: Encapsulated MSC in gellan gum hydrogel show cell viability in vitro and signs of chondrogenesis in mouse subcutaneous implant [387]. In vitro: Gellan gum hydrogels reinforced with nanocellulose demonstrated AF biomechanical properties and bovine AF cell support [388].
Hyaluronan (hyaluronic acid)	+ Retains water, non-immunogenic, anti-inflammatory, and low cost. Bioactive by binding with cell surface receptors and ECM proteins, which promotes cell infiltration [358]. − Osteogenic properties and cytotoxic at high concentrations [361].	In vivo: MSC injected into rat IVD using 15% hyaluronic acid hydrogel. Initial significant cell loss followed by proliferation. An increase in disc height was shown [389]. In vivo: Injection of hyaluronic acid hydrogel in rat tail disc degeneration model demonstrating signs of pain marker reduction and attenuation of inflammation [390]. Clinical: Prospective, multicenter, double-blinded, controlled phase 2 study. Safety and efficacy assessment of allogenic MSC injected with hyaluronic acid in disc degeneration patients (no results posted) [391]. Composites: Ex vivo: Bovine NP cells cultured in a fibrinogen-hyaluronic acid-based hydrogel showed maintenance of some NP markers and disc height recovery in ex vivo organ culture [392].
**Synthetic Hydrogels**		
**Type**	**Material** **Biomechanical Properties**	**IVD Studies**
Poly N-isopropyl-acrylamide (pNIPAM)-based hydrogels	Laponite crosslinked pNIPAM-co-DMAc:	
	+ Thermo-responsive hydrogel injectable above body temperature and solidifies upon cooling to 37 degrees C. [393], supports differentiation of human MSC into NP cells [394], excellent biocompatibility [395]	In vitro: Assessment of hMSC to NP cell differentiation in pNIPAM hydrogel in normoxia and hypoxia [394]. Ex vivo: Human MSC and bovine NPs encapsulated in pNIPAM hydrogel and injected into papain-induced bovine disc degeneration model [393]. In vitro: Laponite cross-linked pNIPAM-co-DMAc encapsulation of hMSC showed NP differentiation was not affected by catabolic culture conditions [356]. In vitro/ex vivo: HA-pNIPAM hydrogel seeded with autologous human NP cells and implanted in intact human IVD explant, demonstrating matrix synthesis [337].
	HA-pNIPAM hydrogel:	
	+ Solidifies beyond 32 °C and is injectable at room temperature [130]	In vitro/ex vivo: Improved NP-like differentiation of hMSC in vitro in HA-pNIPAM hydrogel with GF compared to alginate hydrogel. Direct implantation of hMSC/HA-pNIPAM into bovine organ culture better than pre-differentiating hMSC [396]. In vitro/ex vivo NP cell support and ECM deposition in HA-pNIPAM hydrogel compared to alginate beads, in vitro. Implanted cell-hydrogel construct in bovine disc organ culture showed cell viability [397].
Polyethylene glycol (PEG)-based hydrogels	+ Non-cytotoxic, easily synthesized, PEG-based hydrogels have high hydration properties. Photo-polymerizable composites [358]. − Low biorecognition of cells, which affects cell adhesion properties, non-biodegradable [358].	Composites: In vitro: PEG-hyaluronic acid hydrogel screen for porcine AF and NP cell proliferation and sGAG production identified lower molecular weight hyaluronic acid gels were most suitable for the IVD [398]. In vitro: Porcine NP cell 2D and 3D culture in photo-crosslinked PEG-laminin 111 hydrogel showed support of cell viability and matrix deposition [399]. In vitro/in vivo: PEG-albumin hydrogel tested with human disc cells by comparing 2D, 3D, and mouse subcutaneous implant culture. Significantly higher SOX9 and HAS expression but not of aggrecan or collagen types I/II [400]. Ex vivo: Injection of the photo-polymerizable PEG dimethacrylate nano-fibrillated cellulose composite hydrogel into a bovine organ model of IVD resulting in disc height restoration [401]. In vitro: Bovine NPs cultured in high-molecular-weight hyaluronic acid cross-linked with PEG-amine showed reduced pro-inflammatory markers [402].
Polyvinyl alcohol (PVA)-based hydrogels	+ Mechanical properties easily tailorable to IVD via PVA concentration adjustments [403,404].− Non-degradable and expensive, limited biological testing for IVD applications [361].	In vitro: PVA cryogel biomechanical testing found 3% PVA concentration with 3 freeze-thaw cycles was optimum for mimicking compression properties of the NP [404]. In vitro: Elastic modulus similar to native articular cartilage was attained from the fabrication of PVA and bacterial cellulose nanofiber nanocomposite however, not directly compared to IVD biomechanical properties [405]. Composites: In vitro: PVA containing laponite and bacterial cellulose nanocomposites were mechanically tested showing tailorable stiffness. Wear and fatigue properties were enhanced with nanofiller adjustments and two-component PVA hydrogel could be tailored to mimic IVD compression properties [406]. Ex vivo: PVA-polyvinyl pyrrolidone composite hydrogel showed good fatigue properties and restored compressive stiffness in human cadaver models [403]. In vitro: PVA-silk composite cyrogel showing silk improved cell adhesion and survival of rabbit adipose stem cells over culture period. No proliferation was observed or capacity to encourage NP differentiation [407].
Self-assembling peptide hydrogels (SAPH)	+ Provide the advantages of natural and synthetic biomaterials while overcoming their individual disadvantages, easily tunable biomechanics via peptide sequence modifications, biodegradable and biocompatible, chemically defined, self-healing [408,409]. + Functionalization: Functionalize the peptide with motifs that replicate useful biological molecules, e.g., BMP [410]. Graphene incorporation can act as delivery vehicles for biological factors [411]. Graphene oxide is biocompatible and promotes cell adhesion [412]. − Stability and variable immunogenic concerns remain a challenge [408].	In vitro: Good cell viability of 3D cultured, de-differentiated human NP cells in FEFEFKFK SAPH. NP phenotype (except aggrecan) and GAG synthesis was significantly higher at days 7 and 14 compared to day 1 of 3D culture [413]. In vitro: 3D culture of rabbit NP cells in KLD-12 SAPDH demonstrating increased cell viability and GAG release into media compared to hydrogel only control [414]. In vitro: Rabbit NP cells showed greater cell viability, inward migration, and ECM synthesis in RLN functionalized RADA16 compared with pure RADA16 [415]. In vitro: Human degenerated NP cells 3D cultured in RKP (BMP7 motif) functionalized RAD16-I SAPH showed increased migration, proliferation, and expression of NP marker genes compared to RADA16-I alone [416]. In vitro: Graphene oxide flakes incorporated into FEFEFKFK self-assembling peptide hydrogel mechanically similar to NP and supports bovine NP cells [417].
**Scaffolds for IVD TE**		
**Natural Polymers**		
**Type**	**Material** **Biomechanical Properties**	**IVD Studies**
Silk fibroin	+ Compressive and tensile strength, slow degradation, cytocompatible, modifiable with covalent attachment of additional peptides [360].	In vitro: Bovine AF cells seeded onto porous silk RGD-modified scaffolds demonstrating cell support and ECM deposition and higher collagen II and aggrecan expression than nonmodified scaffold [418]. In vitro: Porcine AF and chondrocyte cells seeded onto biphasic silk scaffold for AF and fibrin/hyaluronic acid for the NP. Increase in GAG and collagen over four-week culture [419]. In vitro/in vivo: Porcine AF cells and hMSC show cell viability and appropriate differentiation toward AF phenotype on multi-layered silk scaffold compared to native AF cells. Subcutaneous mouse implant showed negligible immune response [420].
Alginate	+ Biocompatible, biodegradable, anti-microbial, cheap, high porosity, support cell adhesion and growth [360]. − Poor native mechanical strength needs to be overcome with cross-linking strategies [421].	Composites: In vitro: Alginate-chitosan scaffold showed fiber alignment similar to AF and supports canine AF cell growth and ECM (collagen, aggrecan) deposition [422]. Human NP cells cultured on alginate scaffold demonstrated a fall in cell number over the 21-day culture period [423].
Atelo- collagen	+ Low-immunogenic derivative of collagen, safe and biocompatible, supports stem cell and disc cell regeneration of the IVD [424].	In vitro: Rabbit NP cells seeded on atelocollagen types I and II scaffolds supplemented with BMP demonstrating anabolic gene and protein expression in type II but not type I scaffolds, compared with control [424]. In vivo: Rabbit AF cells cultured on atelocollagen honeycomb-shaped scaffold and transplanted into rabbits showed cell proliferation and production of hyaline-like cartilage [425].
**Synthetic Polymers**		
**Type**	**Material biological and mechanical properties**	**IVD Studies**
Poly-urethane (PU)	+ Biocompatible, biodegradable, decomposes to water and carbon dioxide and high biomechanical properties [358].	In vivo: PU mass transfer device transplanted into punctured porcine AF showed similar biomechanical properties to control group as well as enhanced energy production [426]. Ex vivo: Implantation of biphasic PU scaffold into nucleotomy bovine whole organ culture model demonstrated restoration of disc height, cytocompatibility with native cells, and downregulation of catabolic and upregulation of anabolic genes [427].
Polylactic acid polyglycolic acid (PGA) Copolymer: polylactic-co-glycolic acid (PLGA)	+ Biocompatible and biodegradable, approved by FDA for in-human use [358]. − Potentially encourages nerve in-growth in the disc [428].	In vivo: Sheep AF cells seeded onto PLGA scaffold and implanted in mice demonstrating collagen I expression [429]. Composites: In vitro: Human MSC cultured on a biphasic polyL-lactic acid nanofibrous outer scaffold and inner hyaluronic acid hydrogel to mimic the architecture of AF and NP, respectively. Increased IVD ECM protein accumulation in both regions over 28d culture period [430]. In vivo: PLGA-fibrin gel plugs implanted into empty disc defects resulting in increased nerve ingrowth than empty disc controls [428].
Poly D,L-lactide (PDLLA)	+ Supports adhesion, infiltration, and proliferation of MSC [431]. − Has been shown to encourage osteogenic differentiation of MSC [431].	In vitro: Human AF cells cultured within PDLLA/Bioglass^®^ foam demonstrated proliferation over 4-week culture period and increased PG and collagen deposition than no Bioglass^®^ foam control [432]. Composites: In vitro: b-TCP and calcium carbonate particles loaded into acrylic-terminated oligo[D,L-lactide-co-(ε-caprolactone)]. Biomechanical tests were performed, demonstrated that the addition of fillers aided achieving properties similar to the IVD [433].
Poly-ε-caprolactone (PCL)	+ Biodegradable, FDA approved for in-human use, controlled decomposition time through alternative polymer combinations, high elasticity [358].	In vitro: Electrospun PCL scaffold (AF) combined with cell-seeded hydrogel for rat disc replacement showed effective cell infiltration [434]. In vitro: Bovine MSC seeded onto nanofibrous anistropic PCL scaffold demonstrating collagen deposition and alignment comparable to native bovine AF [435]. In vitro: PCL fibrous scaffold fabricated by wet-spinning showed rabbit AF cell adherence, proliferation, and increased collagen and aggrecan expression over 3-week culture [436].

**Table 2:** Publicly available databases on signaling pathways and protein–protein interactions.

Title	Content	Size	Address
KEGG	Integrated database resource consisting of 18 databases including systems, genomic, chemical, and health information on the molecular interaction networks in biological systems	KEGG Pathway: 536 pathways	https://www.genome.jp/kegg/ [466]
Reactome	Pathway database with interactive web visualization tool	2272 pathways, 10,833 proteins, 12,505 interactions	https://reactome.org/ [467]
STRING	Protein–protein interaction networks	5000 organisms, 24.6 mio proteins, >2000 mio interactions	https://string-db.org/ [468]
WikiPathways	Pathways of different species stored in wiki format	2785 pathways, 28 species	https://wikipathways.org/ [469]
Pathway Commons	Biological pathway data extracted from various databases with visualization tool	4700 pathways, 2.3 mio interactions	https://pathwaycommons.org/ [470]
Omnipath	Literature-curated mammalian signaling pathways from >50 databases	10,934 proteins, 53,542 interactions	http://omnipathdb.org/[471]
MatrixDB	Database focused on interactions established by extracellular matrix proteins, PG and polysaccharides	106,453 associations from 38,921 experiments	http://matrixdb.univ-lyon1.fr/ [472]

## Abbreviations

ADAMTS	A disintegrin and metalloproteinase with thrombospondin motifs
AF	Annulus fibrosus
BMP	Bone morphogenetic protein
CEP	Cartilage edplate
DD	IVD degeneration
ECM	Extracellular matrix
ER	Endoplasmatic recticulum
ERK	Extracellular signal-regulated kinase
FA	Focal adhesion
FGF	Fibroblast growth factor
GAG	Glycosaminoglycan
GDF	Growth differentiation factor
GF	Growth factor
HIF	Hypoxia inducible factor
IGF	Insulin-like growth factor
IL	Interleukin
IVD	Intervertebral disc
JNK	c-Jun NH2terminal kinase
LBP	Low back pain
MAPK	Mitogen-activated protein kinase
MMP	Metalloproteinase
MP	Microparticle
MSC	Mesenchymal stromal cell
mTOR	Mammalian target of rapamycin
NF-κB	Nuclear factor kappa B
NGF	Nerve growth factor
NP	Nucleus pulposus
p38	p38 MAPK
PCL	Poly-ε-caprolactone
PDGF	Platelet-derived growth factor
PDLLA	Poly D,L-lactide
PEG	Polyethylene glycol
PG	Proteoglycan
PGA	Polyglycolic acid
PKN	Prior-knowledge-network
PLGA	Polylactic-co-glycolic acid
PNIPAM	Poly N-isopropylacrylamide
PU	Polyurethane
TE	Tissue engineering
TGF	Transforming growth factor
TNF-α	Tumor necrosis factor alpha
TonEBP	Tonicity-responsive enhancer binding protein
TonEBP/NFAT5	Tonicity-responsive enhancer binding protein/nuclear factor of activated T-cells 5
TRP	Transient receptor potential
